# Detection and prognostic relevance of circulating tumour cells (CTCs) in Asian breast cancers using a label-free microfluidic platform

**DOI:** 10.1371/journal.pone.0221305

**Published:** 2019-09-25

**Authors:** Yoon-Sim Yap, Man Chun Leong, Yong Wei Chua, Kiley Wei Jen Loh, Guek Eng Lee, Elaine Hsuen Lim, Rebecca Dent, Raymond Chee Hui Ng, John Heng-Chi Lim, Garima Singh, Angela Tan, Guofeng Guan, Andrew Wu, Yi Fang Lee, Ali Asgar S. Bhagat, Darren Wan-Teck Lim

**Affiliations:** 1 Division of Medical Oncology, National Cancer Centre Singapore, Singapore; 2 Biolidics Ltd, Singapore; 3 Department of Pathology, Singapore General Hospital, Singapore; 4 Institute of Molecular and Cell Biology, A*Star, Singapore; 5 Clinical Trials and Epidemiology Office, National Cancer Centre Singapore, Singapore; Hunter College, UNITED STATES

## Abstract

**Objectives:**

We aimed to study the prevalence of CTCs in breast cancer (BC) patients undergoing neoadjuvant or palliative therapy with a label-free microfluidic platform (ClearCell FX), and its prognostic relevance in metastatic BC (mBC).

**Materials and methods:**

Peripheral blood samples were collected from 108 BC patients before starting a new line of treatment (“baseline”), majority of whom had mBC (76/108; 70.4%). CTCs were retrieved by dean flow fractionation that enriched for larger cells, and enumerated using immunofluorescence-based staining. Progression-free survival (PFS) in mBC patients was analysed using Kaplan-Meier method; cox proportional hazard models were used for univariable and multivariable analyses.

**Results:**

The detection rate of CTCs before starting a new line of treatment was 75.9% (*n* = 108; median: 8 CTCs/7.5 ml blood) at a cut off of ≥2 CTCs. PFS was inferior for mBC patients with baseline CTC count ≥5 CTCs/7.5 ml blood vs. those with < 5 CTCs/7.5 ml blood (median PFS: 4.3 vs. 7.0 months; *p*-value: 0.037). The prognostic relevance of CTCs was most significant in patients with HER2^-^ mBC (median PFS: 4.1 vs. 8.3 months; *p*-value: 0.032), luminal (HR+HER2-) subtype (median PFS: 4.2 vs. 8.3 months; *p*-value: 0.048), and patients who had one or more prior treatments (median PFS: 4.2 vs. 7.0 months; *p*-value: 0.02). On multivariable analysis, baseline CTC level (hazard ratio (HR): 1.84, *p*-value: 0.02) and pre-treatment status (HR: 1.87, *p*-value: 0.05) were independent predictors of PFS.

**Conclusions:**

This work demonstrates the prognostic significance of CTCs in mBC detected using a label-free size-based enrichment platform.

## Introduction

Breast cancer (BC) is a heterogeneous disease with varying prognosis. Long-term patient outcomes are influenced by biological risk factors such as cancer subtypes, the extent and site of metastatic lesion, and the level of circulating tumour cells (CTCs) [1, 2]. The landmark study by Cristofanilli et al demonstrated that epithelial cell adhesion molecule (EpCAM)-positive CTCs serve as an independent prognostic marker in metastatic BC (mBC); the presence of ≥ 5 CTCs/7.5 ml blood before and after the initiation of therapy was associated with significantly reduced progression-free survival (PFS) and overall survival (OS) [2]. Subsequent studies further confirmed that high CTC levels in patients who had undergone treatment predicted the risk of metastatic spread [3, 4]. The prognostic power of CTCs in BC varies with cancer subtypes, with CTCs having the least prognostic relevance in the HER2^+^ subtype [2, 5].

Tumour heterogeneity and temporal changes in tumour characteristics with clonal selection may influence clinical management. However, serial biopsies of primary and/or metastatic lesions are often limited by the invasive nature and risks of the biopsy procedures. Being minimally invasive, CTC analysis may provide a means to serially monitor tumour evolution [6–10] and potentially direct therapeutic interventions. For example, the DETECT III trial uses HER2^+^ CTCs to test the benefit of adding lapatinib to standard therapy in patients with HER2^-^ metastatic BC (mBC). The CirCe T-DM1 study selects mBC patients with HER2^-^ tumour for trastuzumab-emtansine treatment based on the presence of HER2^+^ CTCs [11–13].

Studies of CTCs in mBC have predominantly focused on the use of the FDA-approved CellSearch technology. The CellSearch approach utilises immunomagnetic beads, targeting EpCAM antigens on epithelial tumour cells for recovering CTCs. One of the hallmark features in cancer metastasis is epithelial-mesenchymal transition (EMT). EMT has been widely described in BC [14] and elevated level of mesenchymal-like CTCs has been associated with treatment failure [15]. Hence there are limitations to EpCAM-immunoaffinity based technology as it cannot detect the cells which have undergone EMT and have downregulated the epithelial markers and are negative for EpCAM. It can lead to the lower detection of CTCs, including those with non-metastatic and metastatic disease [16–18]. The antibody binding also impedes the use of the enriched CTCs for live cell culturing or xenograft assays.

Over the past decade, different CTC isolation methods have been developed. Some of these systems have demonstrated higher retrieval rate of CTCs [19–21]. In this study, the primary objective was to evaluate the detection rate of CTCs among BC patients in the Asian population, using an antibody-independent inertial-based microfluidic platform [22]. The method enriches for CTCs via dean flow fractionation to separate the larger CTCs from smaller blood cells [19, 23]. Briefly, within a proprietary spiral microfluidic chip, Dean drag forces will cause smaller hematologic cells (leukocytes) to migrate along the Dean vortices towards the inner wall of the spiral channels, then back to outer wall again, while the larger CTCs experience additional strong inertial lift forces that will focus along the microchannel inner wall, thus achieving separation [23, 24]. As no biomarkers (antibodies) were used in the enrichment of the circulating tumor cell population, it allows cells that are undergoing epithelial mesenchymal transition (without EpCAM marker expression) to be separated and detected as CTCs. To ensure the specificity of the identified cell population, we used a cocktail of specific antibodies against EpCAM, cytokeratin (CK) and CD45. The classical definition of CTCs is EpCAM+/CK+/CD45-. In this work we modify this definition of CTCs to include cells that are either CK+/CD45-, EpCAM+/CD45-, and EpCAM+/CK+/CD45-, to increase the sensitivity compared to the traditional antibody-based enrichment methods. We also correlated the CTC level with clinico-pathological factors and their association with disease progression in the patients with mBC. To our knowledge, this was one of the few studies to evaluate the prognostic relevance of CTCs recovered with a label-free platform in BC patients [25–27].

## Materials and methods

### Patient population and characteristics

A total of 108 BC patients who were about to commence a new line of systemic therapy in palliative or neoadjuvant setting, were recruited to this study between April 2014 and June 2016 from outpatient clinics at National Cancer Centre Singapore and from inpatient facilities at Singapore General Hospital. “Baseline” blood samples of BC patients analysed in this study were taken prior to initiation of a new line of systemic therapy, while blood samples from 20 healthy individuals served as negative controls. Informed written consent was obtained from all patients in accordance with the approved procedures under the institutional review board (IRB) guidelines (SingHealth Centralised Institutional Review Board (CIRB) Approval no. 2014/119/B).

### Determination of immunohistochemical subtypes

The determination of estrogen receptor (ER), progesterone receptor (PR) and human epidermal growth factor receptor 2 (HER2) status by immunohistochemistry in this study was based on the latest recommendations by the American Society of Clinical Oncology and the College of American Pathologists.[28, 29] ER/PR-expressing, but HER2-null expressing tumours were classified as luminal subtype (luminal A/B). Tumours with null expression in ER/PR and HER2 were classified as triple-negative subtype. Tumours with positive HER2 expression (regardless of ER/PR status) were classified as the HER2-positive subtype.

### CTC enrichment and immunofluorescence staining

Blood samples were collected in either 10 ml EDTA or STRECK DNA blood collection tubes. 7.5 ml of whole blood was processed for each run. Red blood cells were first removed by brief incubation with the addition of red blood cell (RBC) lysis buffer (G-Bioscience, St. Louis, MO, USA), as per manufacturer’s recommendations. Lysed RBCs in the supernatant were discarded after centrifugation. The nucleated cell pellet was suspended in a ClearCell resuspension buffer prior to CTC enrichment on ClearCell FX system, conducted in accordance with manufacturer’s instructions.

Immunofluorescence staining was used to enumerate CTCs, following the definition of cytokeratin and/or EpCAM positivity, CD45 negativity, and presence of nuclei. Enriched CTC sample outputs were transferred onto glass slides and air dried. Air-dried samples were fixed with 4% paraformaldehyde, and blocked with FcR blocking reagent and goat serum. A cocktail of primary antibodies targeting cytokeratin 8, 18, 19 (Miltenyi Biotech), pan-cytokeratin (C11 clone), EpCAM (Cell Signaling Tech), CD45 (Cell Signaling Tech) and DAPI (Invitrogen) was then used to label the cells. Fluorescence-conjugated antibodies (Life Technologies) were used to label the primary antibodies. Automated image acquisition was performed on an epifluorescence microscope, equipped with a motorised stage. A nucleated cell with positive expression of either EpCAM or cytokeratin and negative CD45 expression was classified as a circulating tumour cell.[10] A schematic of the workflow is illustrated in S1 Fig.

### Establishment of system performance

MCF7 and H1975 cells were used for establishing the baseline performance of the ClearCell FX CTC enrichment system at varying tumour cell concentrations. Tumour cells were pre-labelled with Celltracker Orange dye, and counted prior to the introduction into whole blood samples for enrichment on ClearCell FX system. Enriched samples were visualised on a 96-well plate to evaluate the recovery rate. The recovery rate for MCF7 cells was on average ~64.5 ± 12.34% across a cell spiking level from 50 cells/7.5 ml blood to 2000 cell/7.5 ml blood (S2A Fig). Recovery rate for H1975 cells was 69.9 ± 9.74% (S2B Fig).

### CTC analysis

The personnel performing the CTC analysis was blinded to the clinico-pathological status and progression information. Within the mBC patient cohort, CTC counts were assessed according to the number of lines of previous systemic therapy, patient’s age, hormone receptor status (ER/PR), tumour HER2 status, and presence of visceral metastasis. PFS was defined as the time from treatment baseline to disease progression (radiologically or clinically as determined by the treating physician) or death from any cause, whichever came first.

### Statistical analysis

Statistical analysis of the data was performed using Stata software version 14.2 (Stata Corp., College Station, TX, USA). Statistical significance was defined as *p* < 0.05. Given the exploratory nature of our study, the sample size was based on feasibility and accrual over a 2-year period rather than on formal statistical criteria. We planned to enrol at least 100 BC patients, with a minimum of 70 mBC patients evaluable for disease progression. Log-rank testing was applied to compare survival curves while hazard ratio was estimated using the Cox proportional-hazards regression analysis. Univariable and multivariable cox regression analyses of prognostic factors were performed with p-values based on Wald test. CTC counts of breast cancer patients and healthy donors were considered for establishment of the Receiver operating curve (ROC) (S3 Fig) using Graphpad Prism version 5.0 (GraphPad Software, San Diego, CA, USA). Maximal total value of specificity and sensitivity, or Youden’s index was used as the criterion to determine optimal CTC cut-off value (S1 Table).

## Results

### Patients’ characteristics and CTCs prevalence at treatment baseline

For this study, 108 breast cancer patients were recruited (Table 1). The median age of patients enrolled was 56 years (range: 29~75 years). The majority (76/108; 70.4%) of the BC patients had mBC, who were either de novo stage 4 breast cancer cases (37/76) or had relapsed after previous diagnosis of non-metastatic breast cancer (39/76); while 29.6% of the BC patients had non-metastatic breast cancer (nmBC) disease.

**Table 1:** Prevalence of circulating tumour cells at treatment baseline.

Characteristics	Number of subjects	Number of circulating tumour cells (/7.5 ml blood)				
		≥ 2	≥ 5	≥ 10	≥ 50	≥ 100
		Percentage of subjects (%)				
Healthy donors	20	5	0	0	0	0
Stage II/III breast cancer patients (nmBC)	32	81.3	59.4	50.0	21.9	3.1
Stage IV breast cancer patients (mBC)	76	73.7	57.9	35.5	14.5	7.9
**Clinico-pathological characteristics of mBC patients**						
**Prior Line(s) of therapy**						
0 (Treatment naïve)	18	83.3	66.7	50.0	22.2	11.1
1 or more (Pre-treated, including adjuvant/ neo-adjuvant)	58	70.7	55.2	31.0	12.1	6.9
**Hormone receptor status (ER/PR)**						
ER and/or PR +	56	78.6	62.5	39.3	17.9	8.9
ER and PR -	20	60.0	45.0	25.0	5.0	5.0
**Tumour molecular subtype**						
“Luminal” (HR+HER2-)	45	77.8	60.0	40.0	22.2	11.1
Triple negative (HR-/HER-)	13	61.5	53.8	30.8	7.7	7.7
HER2+ (HER2+)	18	72.2	55.6	27.8	0.0	0.0
**HER2/neu status**						
Positive	18	72.2	55.6	27.8	0.0	0.0
Negative	58	74.1	58.6	37.9	19.0	10.3
**Visceral metastases**						
Present	60	78.3	61.7	38.3	15.0	6.7
Absent	16	56.3	43.8	25.0	12.5	12.5

ER, estrogen receptor; PR, progesterone receptor; HER2, human epidermal growth factor receptor 2.

**Fig 1:**
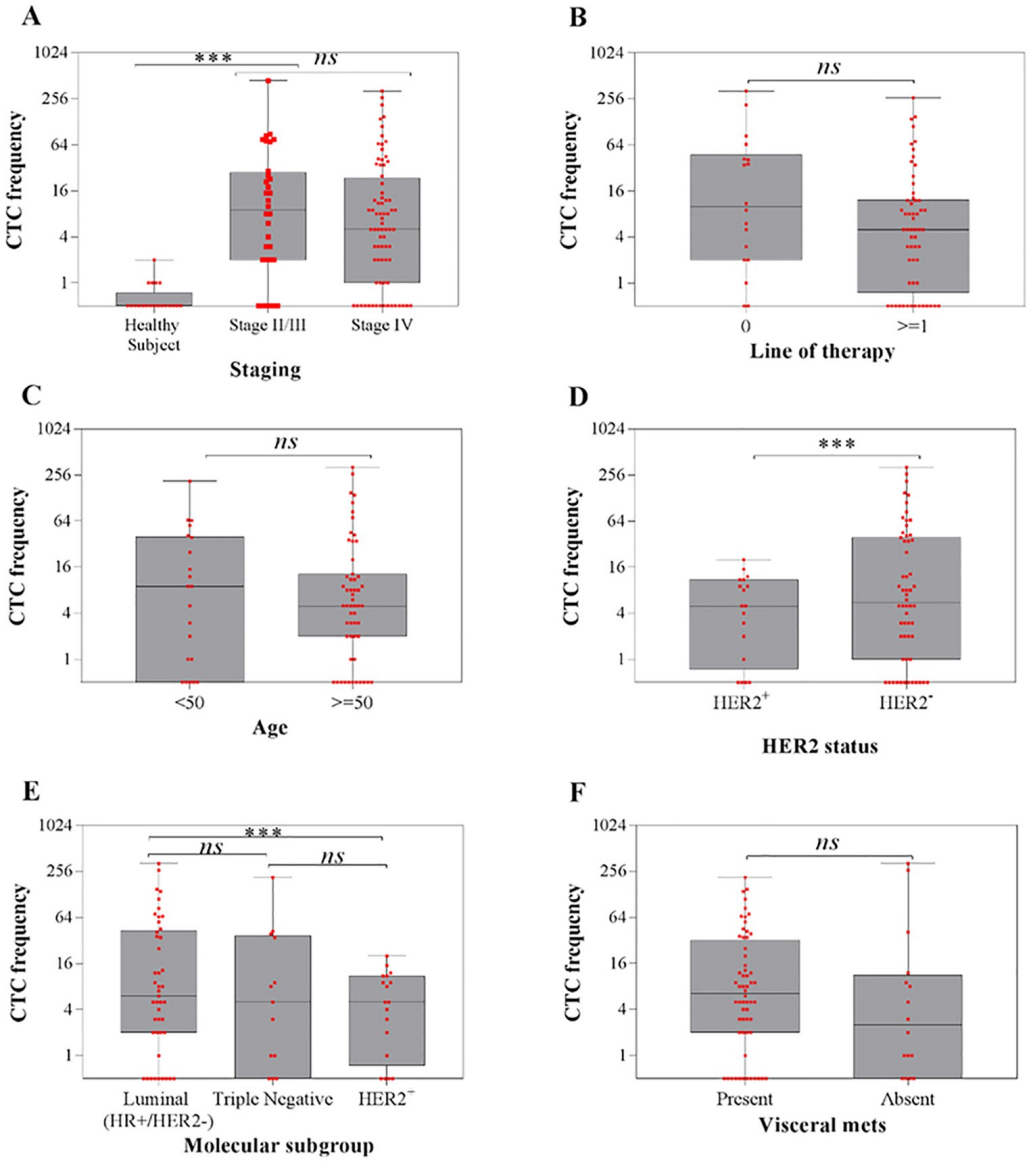
Prevalence of CTCs counts in different categories. (A) CTC counts observed in breast cancer patients and healthy subjects (B-F) CTC counts among metastatic breast cancer patients, stratified according to various independent prognostic factors (B) line of previous therapy, (C) patient age, (D) tumour HER2 status, (E) molecular subgroup and (F) presence of visceral metastases. The horizontal line in box-plot depicts median value. P-value < 0.05 are considered for statistical significance (***), while “ns” denotes non-statistical significance.

Putative CTCs were rarely detected in blood samples from healthy individuals (n = 4 for 1 CTC and n = 1 for 2 CTCs), and were significantly lower in comparison to BC patients (median: 0; range: 0–2 CTCs/7.5 ml blood; *p*-value < 0.0001) (Fig 1A). Receiver operating curve (ROC) analysis was applied to determine CTC cut-off for discriminating healthy individuals and BC patients (*n*: 108). With an optimal cut-off of 2 CTCs/7.5 ml blood, we observed an assay sensitivity of 75.9% and specificity of 95% (S1 Table).

CTCs were detected in 73.7% (56/76) of patients with mBC (median: 5 CTC; range: 0–324 CTC/7.5 ml blood), and 81.3% (26/32) of patients with nmBC (median: 8 CTC; range: 0–441 CTC/7.5 ml blood), using cut-off of 2 CTCs/7.5 ml blood. The nmBC patients consisted mainly of patients with locally advanced breast cancers (clinical stage 3; 24/32, 75%), which could harbour micrometastatic disease and required neoadjuvant chemotherapy for down-staging before surgery. No significant difference in mean CTC values was observed between the nmBC and mBC patient cohorts (*p*-value: 0.646) (Fig 1A).

Within the mBC patient cohort, CTC counts were assessed according to known prognostic factors: the number of lines of previous systemic therapy (including adjuvant/neoadjuvant), patient’s age, tumour HER2 status, molecular subgroups and presence of visceral metastasis (Fig 1B–1F). Tumour HER2 status was observed to be the only significant factor affecting CTC count in mBC patients (Fig 1D). Average CTC count was higher among patients with HER2^-^ tumour subtype (34 CTCs/7.5 ml blood, *n*: 58) compared to patients with HER2+ tumour subtype (regardless of hormone receptor status) (6 CTCs/7.5 ml blood, *n*: 18) (*p*-value, 0.002) (Fig 1D). More patients with HER2-subtype (11/58) had extremely high CTC counts (≥ 50 CTC/7.5 ml blood) as opposed to patients with HER2+ tumour (0/18). Within the HER2- subtypes, there was no significant difference in CTC counts between the triple negative subtype (hormone receptor (HR)-/HER2-) and the luminal subtype (HR+/ HER-) (Fig 1E). However, there was a significant difference in CTC counts between the luminal subtype (mean 36.2 CTCs/7.5 ml blood) and HER2+ subtype (mean 6.4 CTCs/7.5ml blood) (*p*-value, 0.005) (Fig 1E).

### Prognostic relevance of CTC enumeration in mBC patients

**Fig 2:**
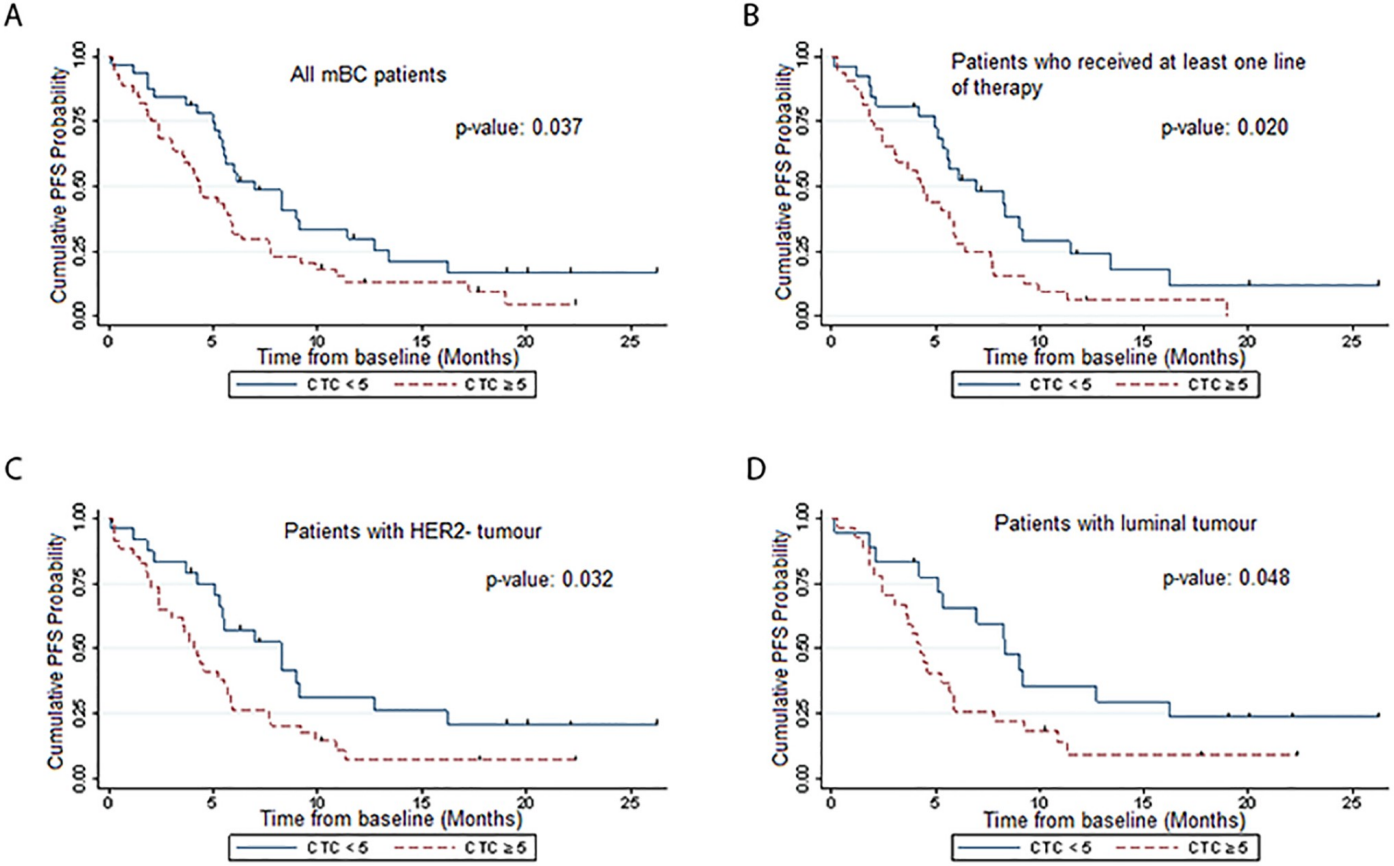
Kaplan- Meier analysis of CTCs levels and progression-free survival. Kaplan-Meier curve showing progression-free survival estimates in mBC patients with < 5 CTC/7.5 ml blood and those with ≥ 5 CTC/7.5 ml blood in (A) all mBC patients, (B) mBC patients who have received at least one line of therapy, (C) mBC patients with HER2^-^ tumour and (D) mBC patients with luminal tumour.

For analysis of progression-free duration, the mBC patient cohort had a median follow- up time of 85 weeks (range: 18 weeks- 130 weeks) from baseline. Baseline CTC counts identified mBC patients who were more likely to experience disease progression (Table 2). Patients with baseline CTCs above the threshold ≥ 5 CTCs/7.5 ml blood (4.3 months) had a shorter time to progression than those with < 5 CTCs/7.5 ml blood (7.0 months) (*p*-value: 0.037) (Fig 2A).

**Table 2:** Progression-free survival among patients with metastatic breast cancer according to CTC level.

	No of patients	Patients with ≥ 5 CTC (%)	Progression-free survival (median)		Hazard ratio	*p*-value (log rank)
			< 5 CTC	≥ 5 CTC		
**All mBC patients**	76	44 (57.9)	7.0	4.3	1.71 [1.03–2.84]	**0.037**
Age						
< 50	21	12 (57.1)	5.6	4.3	1.84 [0.63–5.40]	0.260
≥ 50	55	32 (58.2)	8.3	4.2	1.63 [0.92–2.92]	0.093
**Prior Line(s) of therapy**						
0 (Treatment naïve)	18	12 (66.7)	6.0	4.3	1.56 [0.48–5.09]	0.455
1 or more (Pretreated, including adjuvant/ neo-adjuvant)	58	32 (55.2)	7.0	4.2	1.94 [1.10–3.43]	**0.020**
**Molecular subgroup**						
“Luminal” (HR+HER2-)	45	27 (60.0)	8.3	4.2	1.97 [0.99–3.92]	**0.048**
Triple negative	13	7 (53.8)	5.4	2.4	1.41 [0.37–5.32]	0.609
HER2+	18	10 (55.6)	6.0	6.0	0.85 [0.30–2.45]	0.769
**HER2/neu status**						
HER2+	18	10 (55.6)	6.0	6.0	0.85 [0.30–2.45]	0.769
HER2-	58	34 (58.6)	8.3	4.1	1.90 [1.05–3.46]	**0.032**
**Visceral metastases**						
Present	60	37 (61.7)	6.1	4.3	1.74 [0.97–3.13]	0.062
Absent	16	7 (43.8)	8.3	5.3	1.52 [0.51–4.53]	0.454

ER, estrogen receptor; PR, progesterone receptor; HER2, human epidermal growth factor receptor 2; CTC, circulating tumour cells.

We also examined the prognostic impact of baseline CTCs in patients with different age groups (<50 vs. ≥50), prior treatment status, breast cancer subtypes and visceral metastases status. There was a general trend of inferior PFS in patients with ≥ 5 CTCs/7.5ml blood than those with < 5 CTCs/7.5ml blood for most subgroups except patients with HER2+ tumours.

For prior treatment status, we stratified the mBC patients into treatment naïve patients and pre-treated patients who had received one or more prior systemic treatments (any chemotherapy, targeted therapy or endocrine therapy), in the adjuvant, neo-adjuvant and palliative settings. Baseline CTC level had a significant predictive value among pre-treated mBC patients. Previously-treated mBC patients with ≥ 5 CTCs experienced worse PFS, with median time to progression of 4.2 months vs. 7.0 months for those with < 5 CTCs (*p*-value: 0.02) (Fig 2B).

By stratification of the patients by their HER2 status, we observed a shorter median PFS in HER2^-^ mBC patients with ≥ 5 CTCs vs. < 5 CTCs (4.1 months vs 8.3 months) (*p*-value: 0.032) (Fig 2C). In contrast, CTCs did not predict prognosis among HER2^+^ mBC patients (*p*-value: 0.769). HER2- mBC patients were further stratified into hormone receptor- positive (HR+) luminal subtype and hormone receptor- negative triple negative subtypes. Prognostic impact was observed in the luminal subgroup with worse PFS in the patients with ≥ 5 CTCs/7.5ml blood (*p*-value: 0.048) (Fig 2D). On the other hand, the triple negative subgroup showed non-significant difference (*p*-value: 0.609), possibly due to the small numbers. Patients with visceral metastases also showed a trend towards worse PFS with ≥ 5 CTCs/7.5ml blood (*p*-value: 0.062). In addition, we observed that a higher percentage of patients with visceral metastases had ≥ 5 CTCs than patients without visceral metastases (61.7% vs. 43.8%) (Table 2).

#### Univariable and multivariable analysis of predictors of survival in mBC patients

Various clinico-pathological prognostic factors, including age, baseline CTC count, treatment, breast cancer subtypes, the presence of visceral metastatic sites, and ECOG status were considered for the univariable cox regression analysis in mBC patients. Baseline CTC count was the only significant prognostic factor here (*p*-value: 0.04) (Table 3). However, multivariable analysis of all the factors pointed out baseline CTC count and prior systemic treatment status as significant factors (S2 Table). After variable selection via selection of best subsets using the Akaike Information Criterion (AIC), only baseline CTC counts and prior systemic treatment status remained in the final reduced model (Table 3). Hazard ratios were 1.84 (95% CI: 1.10–3.07, *p*-value: 0.02), and 1.87 (95% CI: 1.00–3.51, *p*-value: 0.0496) for patients with baseline ≥ 5 CTCs/7.5 ml blood and patients undergoing second or subsequent lines of therapy respectively.

**Table 3:** Univariable cox regression analysis, followed by multivariable analysis for progression-free survival in mBC patients.

	*Univariable model*		*Multivariable model*	
Variables	Hazard Ratio (95% CI)	*P-value*	Hazard Ratio (95% CI)	*P-value*
**Age**				
< 50 years	1	-		
≥ 50 years	1.24 (0.69–2.22)	0.468		
**Baseline CTC count**				
CTC < 5	1	-	1	-
CTC ≥ 5	1.71 (1.03–2.84)	0.040	1.84 (1.10–3.07)	0.020
**Line of therapy**				
0 (Treatment naïve)	1	-	1	-
1 or more (Pretreated, including adjuvant/ neo-adjuvant)	1.70 (0.92–3.16)	0.093	1.87 (1.00–3.51)	0.050
**Subtype**				
HER2+	1	-		
Luminal	1.05 (0.59–1.90)	0.861		
Triple negative	1.75 (0.80–3.82)	0.161		
**Metastatic sites**				
Non-visceral	1	-		
Visceral	1.34 (0.73–2.46)	0.351		
**ECOG score**				
0, 1	1	-		
2	0.76 (0.27–2.10)	0.596		

CTC, circulating tumour cells; HER2, human epidermal growth factor receptor 2; ECOG, Eastern Cooperative Oncology Group; CI, confidence interval.

## Discussion

In this prospective study, we determined the prevalence of CTCs among BC patients in an Asian population using an EpCAM-independent CTC recovery platform. CTC detection rates were 81.3% and 73.7% in the nmBC and mBC cohorts respectively. This is generally higher compared to previous studies using EpCAM-dependent platform, which typically detects CTCs in around 12%- 54.5% of the patients in nmBC cohort at cut off of 1 CTC or more [17, 18, 30–33] and 27%-52% in mBC cohort at cut off of 1, 2, or 5 CTCs [2, 3, 33–35]. A study by TA Yap et al showed that using high EpCAM-expressing cell lines, the recovery rates in CellSearch (74±10%) and ClearCell FX (67±11%) were similar (*p* = 0.11), whereas for low EpCAM- expressing cell lines, the recovery rate in ClearCell FX (62 ±8%) was significantly higher (*p*<0.0001) than CellSearch (32±9%)[36]. The heterogeneity of CTCs is compounded by its phenotypic plasticity and epithelial-mesenchymal transition (EMT), which downregulates EpCAM. In order to increase the detection sensitivity of CTCs by immunofluorescence, we utilized two pan-cytokeratin antibodies, in addition to an EpCAM antibody. Despite the enhanced sensitivity, we showed that the specificity of the assay is not compromised. Among the healthy donors, we detected only 1 or 2 circulating epithelial cells in 5 out of 20 donors. These false positives could be due to contaminating skin cells, endothelial cells, rare megakaryocytes, or a weakened leukocyte membrane that would compromise CD45 staining and increased non-specific fluorescence. Nonetheless, we acknowledge that cytokeratins may be limited to epithelial-like CTCs, and the assay might have been more sensitive if we could incorporate mesenchymal markers, such as fibronectin, N-cadherin or SERPINE1 (serpin peptidase inhibitor, clade E) [14].

Our results suggest that baseline CTC count is associated with inferior outcomes, as corroborated in several past studies [2–4, 37]. Results from earlier studies also suggest CTC burden as a predictor of metastatic potential, and potentially a higher visceral tumour burden [4]. Incidentally, we observed a trend of higher median CTC counts among patients with visceral metastasis compared to those without visceral metastasis (Fig 1F).

Despite the association with worse outcomes, the number of CTCs did not correlate with aggressive breast cancer subtypes, HER2+, and triple negative. Instead, the CTC prevalence was significantly higher in the less aggressive HR+/HER2- subtype and had a greater prognostic impact, similar to previous studies using CellSearch platform [2, 5, 38]. One reason could be the often impressive efficacy of anti-HER2 treatment among patients with HER2^+^ tumour, achieving superior survival outcomes in spite of initial high disease volume or CTC count. A second possibility could be that CTCs reflect a transition of the tumour to increased aggressiveness and drug resistance in the HR+/HER2- subtype; for example, by the development of endocrine resistance with *ESR1* mutations [39, 40]. Our data of CTCs having higher prognostic significance in pre-treated patients than treatment naïve patients also suggests an association with drug resistance. Treatment-naïve patients have less resistant disease, and higher probability of longer PFS with first-line treatment in spite of initial CTC count. Lastly, the molecular features of CTCs may have shifted to a mesenchymal or basal-like subtype, which means that the use of epithelial detection markers may underestimate the actual CTC numbers in the triple-negative cohort and HER2+ patients [14, 38, 41].

Univariable and multivariable analyses were further performed to delineate the contribution of confounding prognostic factors in the mBC cohort. Results confirmed those with CTC levels ≥ 5 CTC/7.5 ml blood and patients who had received at least one line of therapy were at higher risk of progression on their systemic therapy (Table 3). Taken together, the observations suggest that we could use baseline CTC count to stratify patients who may respond poorly to standard treatments, and may need more aggressive therapies.

Limitations of our study include the relatively small sample size with heterogeneous patient population and varying intervals of follow-up as per routine clinical practice. Our survival analysis was limited to PFS and not overall survival as only 9 patients had died at the time of last follow- up. Follow-up post-treatment samples were collected from only a subset of patients, hence were not included in this analysis.

In summary, using a dean flow-based separation microfluidic CTC platform, we have demonstrated the prognostic impact of CTCs in mBC. CTC count before treatment is an independent predictor of PFS in patients with HER2-negative mBC. Our overall conclusion is consistent with previous studies which analysed EpCAM^+^ CTC. The findings of our study support the clinical validity of CTCs detected using the label-free microfluidic platform, and potential for other applications.

## Supporting information

S1 Fig Flowchart on CTC enrichment for immunofluorescence. Blood samples were RBC-lysed, and the resultant nucleated cell was suspended in a ClearCell resuspension buffer prior to CTC enrichment on ClearCell FX system. Enriched CTC samples were concentrated and immobilised on a glass slide for immunofluorescence staining and automated image acquisition.
(TIF)

S2 Fig Performance of CTCs enrichment. (A) Tumour cell recovery rate of the entire enrichment workflow as a function of varying concentration of cancer cell (MCF7). (B) Average recovery rate achieved with MCF7 (breast) and H1975 (lung) cell lines. Median value is indicated by a horizontal line on the plots.
(TIF)

S3 Fig Establishing the sensitivity and specificity of the CTC counting assay. Receiver operating curve (ROC) was established based on CTC counts in breast cancer patients and healthy donors.
(TIF)

S1 Table Sensitivity vs specificity table. (DOCX)

S2 Table Multivariable cox regression analysis of prognostic factors for progression-free survival in mBC patients. (DOCX)

S1 Dataset Dataset. (XLSX)
