# Supplementary figures and images for: Detection and prognostic relevance of circulating tumour cells (CTCs) in Asian breast cancers using a label-free microfluidic platform
Source: PLoS One. 2019 Sep 25;14(9):e0221305. doi: 10.1371/journal.pone.0221305 (PMC6760773; doi:10.1371/journal.pone.0221305)

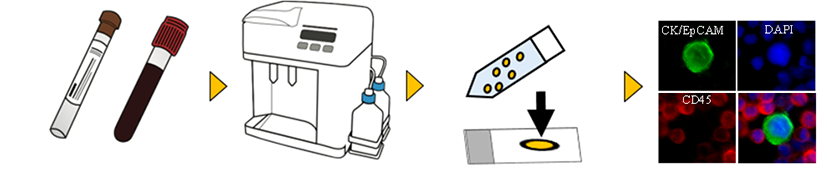

Supplement: S1 Fig — Blood samples were RBC-lysed, and the resultant nucleated cell was suspended in a ClearCell resuspension buffer prior to CTC enrichment on ClearCell FX system. Enriched CTC samples were concentrated and immobilised on a glass slide for immunofluorescence staining and automated image acquisition. (TIF) [file pone.0221305.s001.tif]

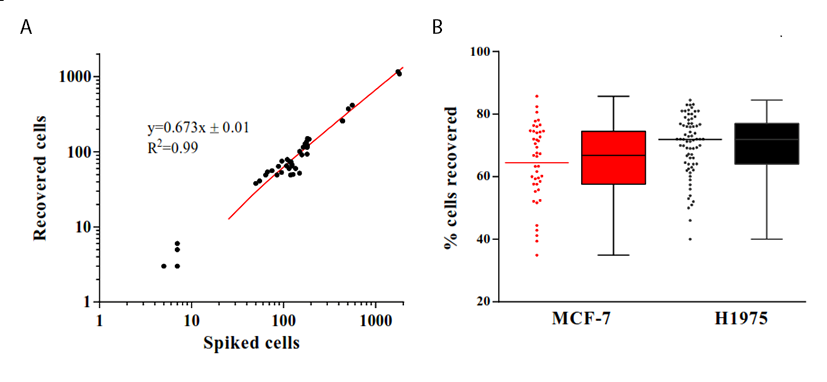

Supplement: S2 Fig — (A) Tumour cell recovery rate of the entire enrichment workflow as a function of varying concentration of cancer cell (MCF7). (B) Average recovery rate achieved with MCF7 (breast) and H1975 (lung) cell lines. Median value is indicated by a horizontal line on the plots. (TIF) [file pone.0221305.s002.tif]

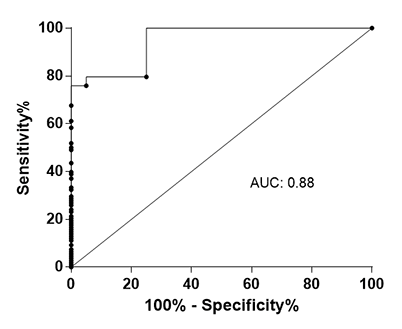

Supplement: S3 Fig — Receiver operating curve (ROC) was established based on CTC counts in breast cancer patients and healthy donors. (TIF) [file pone.0221305.s003.tif]
