# Supplementary material for: Detection and prognostic relevance of circulating tumour cells (CTCs) in Asian breast cancers using a label-free microfluidic platform
Source: PLoS One. 2019 Sep 25;14(9):e0221305. doi: 10.1371/journal.pone.0221305 (PMC6760773; doi:10.1371/journal.pone.0221305)
Supplement: S1 Table — (DOCX) [file pone.0221305.s004.docx]

### S1 Table. Sensitivity vs specificity table

| **Cutoff** | **Sensitivity%** | **95% CI** | **Specificity%** | **95% confidence interval (CI)** | **Likelihood ratio** |
| --- | --- | --- | --- | --- | --- |
| > 0.5 | 79.63 | 70.8% to 86.77% | 75 | 50.9% to 91.34% | 3.185 |
| **> 1.5** | **75.93** | **66.75% to 83.63%** | **95** | **75.13% to 99.87%** | **15.19** |
| > 2.5 | 67.59 | 57.91% to 76.28% | 100 | 83.16% to 100% |  |
| > 3.5 | 61.11 | 51.25% to 70.34% | 100 | 83.16% to 100% |  |
| > 4.5 | 58.33 | 48.45% to 67.75% | 100 | 83.16% to 100% |  |
| > 5.5 | 51.85 | 42.03% to 61.57% | 100 | 83.16% to 100% |  |
| > 6.5 | 50 | 40.22% to 59.78% | 100 | 83.16% to 100% |  |
| > 7.5 | 49.07 | 39.33% to 58.87% | 100 | 83.16% to 100% |  |
| > 8.5 | 43.52 | 34% to 53.4% | 100 | 83.16% to 100% |  |
| > 9.5 | 39.81 | 30.52% to 49.68% | 100 | 83.16% to 100% |  |
| > 10.5 | 38.89 | 29.66% to 48.75% | 100 | 83.16% to 100% |  |
| > 11.5 | 37.04 | 27.94% to 46.86% | 100 | 83.16% to 100% |  |
| > 12.5 | 33.33 | 24.55% to 43.05% | 100 | 83.16% to 100% |  |
| > 14 | 32.41 | 23.72% to 42.09% | 100 | 83.16% to 100% |  |
| > 16.5 | 29.63 | 21.23% to 39.18% | 100 | 83.16% to 100% |  |
| > 19 | 28.7 | 20.41% to 38.2% | 100 | 83.16% to 100% |  |
| > 20.5 | 27.78 | 19.59% to 37.22% | 100 | 83.16% to 100% |  |
| > 22 | 26.85 | 18.78% to 36.24% | 100 | 83.16% to 100% |  |
| > 24 | 25.93 | 17.97% to 35.25% | 100 | 83.16% to 100% |  |
| > 27 | 24.07 | 16.37% to 33.25% | 100 | 83.16% to 100% |  |
| > 32 | 23.15 | 15.57% to 32.25% | 100 | 83.16% to 100% |  |
| > 35.5 | 21.3 | 14% to 30.22% | 100 | 83.16% to 100% |  |
| > 37.5 | 20.37 | 13.23% to 29.2% | 100 | 83.16% to 100% |  |
| > 40 | 19.44 | 12.46% to 28.17% | 100 | 83.16% to 100% |  |
| > 41.5 | 18.52 | 11.69% to 27.14% | 100 | 83.16% to 100% |  |
| > 43.5 | 17.59 | 10.94% to 26.1% | 100 | 83.16% to 100% |  |
| > 50.5 | 16.67 | 10.19% to 25.06% | 100 | 83.16% to 100% |  |
| > 60.5 | 15.74 | 9.445% to 24% | 100 | 83.16% to 100% |  |
| > 65.5 | 14.81 | 8.712% to 22.94% | 100 | 83.16% to 100% |  |
| > 68.25 | 13.89 | 7.987% to 21.87% | 100 | 83.16% to 100% |  |
| > 70.75 | 12.96 | 7.272% to 20.79% | 100 | 83.16% to 100% |  |
| > 71.5 | 12.04 | 6.567% to 19.7% | 100 | 83.16% to 100% |  |
| > 73.5 | 11.11 | 5.875% to 18.6% | 100 | 83.16% to 100% |  |
| > 79.5 | 9.259 | 4.53% to 16.37% | 100 | 83.16% to 100% |  |
| > 86.25 | 7.407 | 3.252% to 14.07% | 100 | 83.16% to 100% |  |
| > 99.75 | 6.481 | 2.645% to 12.9% | 100 | 83.16% to 100% |  |
| > 125.5 | 5.556 | 2.066% to 11.7% | 100 | 83.16% to 100% |  |
| > 144.5 | 4.63 | 1.52% to 10.47% | 100 | 83.16% to 100% |  |
| > 181 | 3.704 | 1.018% to 9.212% | 100 | 83.16% to 100% |  |
| > 238.5 | 2.778 | 0.5765% to 7.904% | 100 | 83.16% to 100% |  |
| > 294 | 1.852 | 0.2251% to 6.53% | 100 | 83.16% to 100% |  |
| > 382.5 | 0.9259 | 0.02344% to 5.051% | 100 | 83.16% to 100% |  |
