# Supplementary material for: Detection and prognostic relevance of circulating tumour cells (CTCs) in Asian breast cancers using a label-free microfluidic platform
Source: PLoS One. 2019 Sep 25;14(9):e0221305. doi: 10.1371/journal.pone.0221305 (PMC6760773; doi:10.1371/journal.pone.0221305)
Supplement: S2 Table — (DOCX) [file pone.0221305.s005.docx]

## S2 Table. Multivariable cox regression analysis of prognostic factors for progression-free survival in mBC patients

| **Variables** |  | **Hazard Ratio**  **(95% C.I.)** | ***P-value*** |
| --- | --- | --- | --- |
| **Age** |  |  |  |
| < 50 years |  | 1 | - |
| ≥ 50 years |  | 1.20 (0.66 – 2.18) | 0.547 |
| **Baseline CTC count** |  |  |  |
| CTC < 5 |  | 1 | - |
| CTC ≥ 5 |  | 1.90 (1.12 – 3.24) | 0.018 |
| **Line of therapy** |  |  |  |
| 0 (Treatment naïve) |  | 1 | - |
| 1 or more (Pretreated, including adjuvant/ neo-adjuvant) |  | 1.94 (1.00 – 3.78) | 0.052 |
| **Subtype** |  |  |  |
| HER2 |  | 1 | - |
| Luminal |  | 1.28 (0.69 – 2.37) | 0.433 |
| Triple negative |  | 2.04 (0.91 – 4.56) | 0.082 |
| **Metastatic sites** |  |  |  |
| Non-visceral |  | 1 | - |
| Visceral |  | 1.07 (0.56 – 2.03) | 0.837 |
| **ECOG score** |  |  |  |
| 0, 1 |  | 1 | - |
| 2 |  | 1.25 (0.43 – 3.70) | 0.681 |
